# Beneficial effects of a Paleolithic diet on cardiovascular risk factors in type 2 diabetes: a randomized cross-over pilot study

**DOI:** 10.1186/1475-2840-8-35

**Published:** 2009-07-16

**Authors:** Tommy Jönsson, Yvonne Granfeldt, Bo Ahrén, Ulla-Carin Branell, Gunvor Pålsson, Anita Hansson, Margareta Söderström, Staffan Lindeberg

**Affiliations:** 1 Department of Clinical Sciences, Lund, Lund University, Box 117, 221 00 Lund, Sweden; 2 Department of Food Technology, Engineering and Nutrition, Lund University, Lund, Sweden; 3 Primary Health Care, Region Skåne, Regionhuset, Baravägen, 221 00 Lund, Sweden; 4 Department of Clinical Sciences, Malmö, CRC, 205 02 Malmö, Sweden

## Abstract

**Background:**

Our aim was to compare the effects of a Paleolithic ('Old Stone Age') diet and a diabetes diet as generally recommended on risk factors for cardiovascular disease in patients with type 2 diabetes not treated with insulin.

**Methods:**

In a randomized cross-over study, 13 patients with type 2 diabetes, 3 women and 10 men, were instructed to eat a Paleolithic diet based on lean meat, fish, fruits, vegetables, root vegetables, eggs and nuts; and a Diabetes diet designed in accordance with dietary guidelines during two consecutive 3-month periods. Outcome variables included changes in weight, waist circumference, serum lipids, C-reactive protein, blood pressure, glycated haemoglobin (HbA1c), and areas under the curve for plasma glucose and plasma insulin in the 75 g oral glucose tolerance test. Dietary intake was evaluated by use of 4-day weighed food records.

**Results:**

Study participants had on average a diabetes duration of 9 years, a mean HbA1c of 6,6% units by Mono-S standard and were usually treated with metformin alone (3 subjects) or metformin in combination with a sulfonylurea (3 subjects) or a thiazolidinedione (3 subjects). Mean average dose of metformin was 1031 mg per day. Compared to the diabetes diet, the Paleolithic diet resulted in lower mean values of HbA1c (-0.4% units, *p *= 0.01), triacylglycerol (-0.4 mmol/L, *p *= 0.003), diastolic blood pressure (-4 mmHg, *p *= 0.03), weight (-3 kg, *p *= 0.01), BMI (-1 kg/m^2^, *p *= 0.04) and waist circumference (-4 cm, *p *= 0.02), and higher mean values of high density lipoprotein cholesterol (+0.08 mmol/L, *p *= 0.03). The Paleolithic diet was mainly lower in cereals and dairy products, and higher in fruits, vegetables, meat and eggs, as compared with the Diabetes diet. Further, the Paleolithic diet was lower in total energy, energy density, carbohydrate, dietary glycemic load, saturated fatty acids and calcium, and higher in unsaturated fatty acids, dietary cholesterol and several vitamins. Dietary GI was slightly lower in the Paleolithic diet (GI = 50) than in the Diabetic diet (GI = 55).

**Conclusion:**

Over a 3-month study period, a Paleolithic diet improved glycemic control and several cardiovascular risk factors compared to a Diabetes diet in patients with type 2 diabetes.

**Trial registration:**

ClinicalTrials.gov NCT00435240.

## Background

While dietary management is a cornerstone in the treatment of type 2 diabetes, high quality data on the efficacy of dietary treatment of type 2 diabetes are lacking, according to a recent Cochrane review [1]. Since nutritional science is hampered by confounders, an evolutionary approach has been suggested. It has been postulated that foods that were regularly eaten during human evolution, in particular during the Paleolithic (the 'Old Stone Age', 2.5–0.01 million years BP), may be optimal for prevention and treatment of type 2 diabetes, CVD and insulin resistance [2,3]. A Paleolithic diet is a modern dietary regimen based on foods presumably eaten regularly during the Paleolithic, which includes lean meat, fish, shellfish, fruits, vegetables, roots, eggs and nuts, but not grains, dairy products, salt or refined fats and sugar, which became staple foods long after the appearance of fully modern humans.

To date, only a few studies have examined the effects of a Paleolithic diet on disease and risk factors for disease. In a randomized controlled study in 29 men with ischemic heart disease (IHD) and impaired glucose tolerance or type 2 diabetes (mean HbA1C 4.8% at baseline), we found improved glucose tolerance independent of weight-loss after 12 weeks of Paleolithic diet compared to a Mediterranean-like diet [4]. In the same study, the Paleolithic diet was reportedly lower in glycemic load (GL) than the Mediterranean-like diet [4]. The clinical relevance of glycemic index (GI) and GL is presently being discussed [5]. Some studies show beneficial effects of a low GI/GL diet on risk factors for CVD in diabetes, while other studies do not [6-8]. In a non-controlled study on 14 healthy individuals, Österdahl et al found that three weeks on a Paleolithic diet significantly reduced weight, BMI, waist circumference, systolic blood pressure (SBP) and plasminogen activator inhibitor-1 (PAI-1) [9]. In another non-controlled study in nine healthy overweight individuals where intervention food was supplied and weight kept steady, Frassetto et al found that ten days of a Paleolithic diet improved diastolic blood pressure (DBP), glucose tolerance, insulin sensitivity and lipid profiles [10]. In a randomized controlled feeding trial in domestic swine, we found higher insulin sensitivity, lower C-reactive protein (CRP) and lower DBP after 15 months of a Paleolithic diet, compared with a cereal-based swine feed [11]. This study also showed a low-grade inflammation of the pancreas in the swine who had eaten a cereal based swine feed [11]. In a non-controlled study of ten Australian Aborigines with diabetes and a mean BMI of 27 kg/m^2^, O'Dea found that reversion to a hunter-gatherer lifestyle during 7 weeks led to 10% weight loss and reductions in fasting and 2 hour glucose and fasting insulin [12]. In a similar study on healthy Australian Aborigines by the same authors, the insulin response to 70 g of starch from white bread was reduced, while the glucose response was not, after 10–12 weeks of reversion to a traditional lifestyle [13]. In an epidemiologic study, we found that traditional Pacific Islanders of Kitava, Papua New Guinea, had no signs of IHD, stroke or markers of the metabolic syndrome, possibly because of their traditional lifestyle [14-16]. Thus, we have previously shown beneficial effects from Paleolithic diet on glycemic control and risk markers for CVD in patients with IHD and in domestic pigs. No study, however, has so far examined the same potential beneficial effect of Paleolithic diet when compared to diabetes diet in subjects with type 2 diabetes.

In the present study, therefore, our aim was to examine the effect on glycemic control and risk factors for CVD of food-based (as opposed to macronutrient based) dietary advice according to this Paleolithic diet model over a 3-month period in patients with type 2 diabetes. The patients were recruited in a primary health care setting, and effects of a Paleolithic diet was compared with effects of dietary advice in accordance with current guidelines for people with diabetes [17].

## Methods

### Patients

Approval of the study was obtained from the regional Medical Ethics Committee and the trial was registered at ClinicalTrials.gov (Identifier: NCT00435240). The study was a randomized, cross-over, dietary intervention study in 13 patients with type 2 diabetes without insulin treatment, 3 women and 10 men, recruited from three primary health care units in the Lund area in Sweden. We included adult patients with type 2 diabetes and a C-peptide value above zero, unaltered medical diabetes treatment and stable weight since three months before start of study, HbA1c above 5.5% by Mono-S standard, creatinine below130 μmol/L, liver enzymes below four times their respective upper reference value, no chronic oral or injection steroid treatment and no acute coronary event or change in medication of beta blockers or thyroxin since six months before start of study. Exclusion criteria during ongoing study were change in beta blocker or thyroxin medication, chronic oral or injection steroid treatment, warfarin treatment, creatinine above 130 μmol/L or liver enzymes above four times their respective upper reference value, acute coronary event, and physical or psychological illness or changes in personal circumstances which would make further study participation impossible.

Recruitment for the study during routine clinical work was performed by TJ, UCB, GP, AH and MS. In addition, a letter containing written study information was sent by TJ to subjects at two of the health stations who from journal data seemed to match the inclusion criteria. All recruited subjects were given oral and written study information prior to signing a consent form to participate in the study and were then further assessed for eligibility.

### Procedure

All eligible subjects were informed of the intention to compare two healthy diets in the treatment of type 2 diabetes and that it was unknown if any of them would be superior to the other. At study start all eligible subjects were randomized to start with either a Diabetes diet in accordance with current guidelines [17] or a Paleolithic diet. Randomization was performed by UCB, GP and AH by opening opaque, sealed envelopes (prepared by TJ) containing a note of the initial diet with equal proportions of envelopes for both diets. After randomization, there was no blinding of dietary assignment to study participants, nor to those administering the interventions or assessing the outcomes. Immediately after randomization, all subjects received oral and written information individually (by UCB, GP or AH) in the morning about their respective initial diet. After three months all subjects switched diets and received new oral and written information individually (by UCB, GP or AH) about the diet of the following three months. Written information with dietary advice and food recipes were similarly formulated for both diets. For increased conformity, the dietary advice and data collection procedure were discussed by all authors except YG at several meetings prior to start of study. Advice about regular physical activity was given equally to all subjects.

The information on the Diabetes diet stated that it should aim at evenly distributed meals with increased intake of vegetables, root vegetables, dietary fiber, whole-grain bread and other whole-grain cereal products, fruits and berries, and decreased intake of total fat with more unsaturated fat. The majority of dietary energy should come from carbohydrates from foods naturally rich in carbohydrate and dietary fiber. The concepts of glycemic index and varied meals through meal planning by the Plate Model were explained [18]. Salt intake was recommended to be kept below 6 g per day.

The information on the Paleolithic diet stated that it should be based on lean meat, fish, fruit, leafy and cruciferous vegetables, root vegetables, eggs and nuts, while excluding dairy products, cereal grains, beans, refined fats, sugar, candy, soft drinks, beer and extra addition of salt. The following items were recommended in limited amounts for the Paleolithic diet: eggs (≤2 per day), nuts (preferentially walnuts), dried fruit, potatoes (≤1 medium-sized per day), rapeseed or olive oil (≤1 tablespoon per day), wine (≤1 glass per day). The intake of other foods was not restricted and no advice was given with regard to proportions of food categories (e.g. animal versus plant foods). The evolutionary rationale for a Paleolithic diet and potential benefits were explained [19].

### Evaluation

An oral glucose tolerance test (OGTT) was performed in the morning after obtaining venous blood samples and measurements of blood pressure, weight and waist circumference in the primary care unit (by UCB, GP or AH) at study start, after 3 months (when switching to a new diet) and at the end of the study (after 6 months). 75 g glucose was ingested. Blood samples for plasma glucose and insulin during OGTT were obtained at 0, 15, 30, 60, 90 and 120 minutes. Changes in the area under the curve (AUC) between 0 and 120 min during OGTT for plasma glucose (AUC Glucose_0–120_) and plasma insulin (AUC Insulin_0–120_) were predefined primary endpoints, along with changes in body weight, waist circumference, serum lipids, CRP, blood pressure and glycated haemoglobin A1c (HbA1c) by Mono-S standard. The base of the AUC was set at 0 mmol/L for glucose and 0 pmol/L for insulin. The stimulated secretion was represented by the areas under the glucose and insulin curves using levels at 0 min as the base of the area. The Homeostatic model assessment (HOMA) was used for assessing beta-cell function (%B) and insulin sensitivity (%S), as percentages of a normal reference population, and insulin resistance (IR, the reciprocal of %S (100/%S)) [20]. Values for %B, %S, and IR were derived from fasting plasma glucose and insulin using the HOMA2 computer model v2.2 [20]. Insulin sensitivity index (ISI_0,120_) was calculated from fasting (0 min) and 120 min (post-OGTT) insulin and glucose concentrations [21]. A 4-day weighed food record on four consecutive days, including one weekend day, with weighing of each food item on a digital weighing scale (that could be set to zero), was completed by the participants, starting 6 weeks after initiating each diet. Nutrient compositions were calculated by YG using data from The Swedish Food Database of the National Food Administration in Sweden. GL and GI for the two diets were calculated. Underlying concept of dietary GL and dietary GI is food GI, introduced by Jenkins et al [22], reflecting the postprandial glucose response after a specific food rich in carbohydrate, expressing the quality of the carbohydrates. Wolever and Jenkins also suggested the possibility of ranking diets based on dietary GI calculated from the proportional GI contribution of the included foods containing carbohydrate [23]. To include also the quantity of carbohydrates consumed GL was introduced by Salmerón et al expressing the glycemic effect of the diet [24]. While dietary GI is expressing the quality of the carbohydrates consumed GL represent both the quantity and the quality of the carbohydrates consumed. Thus, dietary GL in this study was calculated as the result from multiplying available carbohydrate (g) for the food reported by the subjects during the 4-day weighed food record with the specific food's GI divided by 100. Available carbohydrate was based on total carbohydrate minus dietary fibre. Food's GI values (glucose as reference) were taken from the compilation by Foster-Powel et al [25]. Dietary GI was calculated as 100 multiplied with dietary GL divided by the amount of available carbohydrate (g) in the diet.

### Statistics

A pre-study power calculation showed that 15 subjects would be required to detect, with 80% power and at a significance level of 5%, a 15% reduction in AUC Glucose_0–120_. Two-way paired t-test was used to analyze within-subject changes in absolute values, while two-way unpaired t-test was used to analyze between-subject changes in absolute values. All outcome variables showed reasonable normal distribution in normal plots. Within-subject changes in outcome variables after first and second diet and within-subject changes in reported dietary intake during first and second diet were used to check for period effects [26]. Mean values of outcome variables and reported dietary intakes for the group starting with Paleolithic diet was compared with the group starting with Diabetes diet in order to check for carry-over effects [26]. Exploratory analyses were performed on outcome variables with significant effects from the Paleolithic diet as compared to the Diabetes diet. Exploratory analyses consisted of bivariate correlations between within-subject differences (Δ) in outcome and dietary variables. Significantly correlating variables were entered into a stepwise forward linear regression analyses.

## Results

### Recruitment and participant flow

The study started in January 2005 and the last participant was followed up in September 2007 after which the study was stopped. Out of 26 subjects assessed for eligibility, nine were not eligible since they did not meet the inclusion criteria or refused to participate. Out of the remaining 17 eligible subjects, who were all randomized and started on the study, four subjects were excluded for the following reasons: one starting with Paleolithic diet was wrongly included with ongoing warfarin treatment, one starting with Paleolithic diet was unwilling to continue due to abdominal pains and bloating, one starting with Diabetes diet was excluded after developing leukemia, and one starting with Diabetes diet was excluded after developing heart failure. All reported analyses are "per protocol" analyses on the 13 participants who completed the trial.

### Medication

Study participants were on average treated with just above four drugs per day, which usually included metformin alone (3 subjects) or metformin in combination with a sulfonylurea (3 subjects) or a thiazolidinedione (3 subjects)(Table 1, 2). Medication usually also included a lipid lowering drug (8 of 13 study participants and always statin treatment) and more than one anti-hypertensive drug per day (Table 1, 2). All medication remained unchanged during the whole study with the following exceptions:

**Table 1:** Baseline characteristics (mean ± SD)

Sex male/female (n)	10/3
Age (year)	64 ± 6
Diabetes duration (year)	8 ± 5
Diabetic values at OGTT yes/no (n)	12/1
Lipid lowering drug (= statin) yes/no (n)	8/5
Drugs per day	4.3 ± 2.3
Anti-hypertensive drugs per day	1.5 ± 1.5
Beta-blocker yes/no (n)	4/9
Thiazide yes/no (n)	4/9
ACE-inhibitor yes/no (n)	5/8
Angiotensin-II receptor blocker yes/no (n)	4/9
Calcium channel blocker yes/no (n)	3/10
Anti-diabetic drugs per day	1.2 ± 0.9
Metformin yes/no (n)	9/4
Sulfonylurea yes/no (n)	3/10
Thiazolidinedione yes/no (n)	3/10
Metformin per day (mg)	1031 ± 864
HbA1C (%, Mono-S)	6.6 ± 0.6
Cholesterol (mmol/l)	4.4 ± 1.1
LDL (mmol/l)	2.9 ± 0.9
HDL (mmol/l)	1.28 ± 0.22
TG (mmol/l)	1.5 ± 0.7
CRP (mg/l)	2.4 ± 1.8
SBP (mmHg)	150 ± 21
DBP (mmHg)	83 ± 10
Height (cm)	171 ± 5
Weight (kg)	87 ± 17
BMI (kg/m^2^)	30 ± 7
Waist (cm)	103 ± 14
fP-glucose (mmol/l)	7.8 ± 1.2
fP-insulin (pmol/l)	98 ± 44
AUC glucose_0–120 _(mmol/l·min)	1607 ± 218
AUC glucose_0–120 _(mmol/l·min)^1^	667 ± 186
AUC insulin_0–120 _(nmol/l·min)	30 ± 12
AUC insulin_0–120 _(nmol/l·min)^1^	18 ± 8
ISI_0,120 _(mg·l^2^/mmol·mU·min)	40 ± 9
HOMA2 %B	65 ± 34
HOMA2 %S	59 ± 27
HOMA2 IR	2.0 ± 0.8

^1^stimulated secretion, area under curve at OGTT with value at 0 minutes as baseline.

**Table 2:** Baseline differences and carry-over effects between groups with different starting diets (mean ± SD)

	Paleolithic diet first (7 of 13)		Diabetes diet first (6 of 13)		P*	
	Baseline	Individual mean for both diets	Baseline	Individual mean for both diets	Baseline	Carryover effect
Sex male/female (n)	6/1		4/2		0.6	
Age (year)	66 ± 6		63 ± 6		0.3	
Diabetes duration (year)	6 ± 4		11 ± 6		0.13	
Diabetic values at OGTT yes/no (n)	6/1		6/0		1.0	
Lipid lowering drug (= statin) yes/no (n)	4/3		4/2		1.0	
Drugs per day	4.9 ± 2.7		3.7 ± 1.8		0.4	
Anti-hypertensive drugs per day	1.9 ± 1.7		1.2 ± 1.2		0.4	
Beta-blocker yes/no (n)	3/7		1/6		1.0	
Thiazide yes/no (n)	3/7		1/6		1.0	
ACE-inhibitor yes/no (n)	3/7		2/6		1.0	
Angiotensin-II receptor blocker yes/no (n)	2/7		2/6		1.0	
Calcium channel blocker yes/no (n)	2/7		1/7		1.0	
Anti-diabetic drugs per day	0.9 ± 0.9		1.5 ± 0.8		0.2	
Metformin yes/no (n)	4/7		5/6		1.0	
Sulfonylurea yes/no (n)	1/7		2/6		1.0	
Thiazolidinedione yes/no (n)	1/7		2/6		1.0	
Metformin per day (mg)	814 ± 790		1283 ± 950		0.4	
HbA1C (%, Mono-S)	6.2 ± 0.2	5.4 ± 0.5	6.9 ± 0.7	6.2 ± 0.7	0.06	0.04
Cholesterol (mmol/l)	4.2 ± 1.3	4.4 ± 1.4	4.7 ± 0.9	4.3 ± 0.9	0.5	0.9
LDL (mmol/l)	2.7 ± 1.0	2.78 ± 1.25	3.0 ± 0.8	2.67 ± 0.65	0.6	0.8
HDL (mmol/l)	1.28 ± 0.25	1.36 ± 0.27	1.28 ± 0.19	1.24 ± 0.26	1.0	0.4
TG (mmol/l)	1.4 ± 0.5	1.1 ± 0.4	1.7 ± 0.8	1.4 ± 0.7	0.5	0.3
CRP (mg/l)	2.9 ± 2.2	2.2 ± 1.6	1.9 ± 1.3	2.4 ± 1.5	0.4	0.9
SBP (mmHg)	156 ± 23	148 ± 14	144 ± 18	141 ± 17	0.3	0.4
DBP (mmHg)	83 ± 11	80 ± 8	84 ± 9	82 ± 7	0.8	0.6
Height (cm)	172 ± 4		170 ± 6		0.6	
Weight (kg)	82 ± 13	77 ± 11	92 ± 20	88 ± 15	0.3	0.2
BMI (kg/m^2^)	28 ± 4	26 ± 3	32 ± 8	31 ± 6	0.3	0.14
Waist (cm)	97 ± 9	92 ± 8	109 ± 17	101 ± 10	0.2	0.10
fP-glucose (mmol/l)	7.1 ± 0.7	6.6 ± 1.1	8.6 ± 1.2	8.0 ± 1.3	0.02	0.052
fP-insulin (pmol/l)	118 ± 53	64 ± 19	75 ± 12	73 ± 23	0.07	0.5
AUC glucose_0–120 _(mmol/l·min)	1498 ± 227	1321 ± 310	1734 ± 128	1574 ± 289	0.046	0.2
AUC glucose_0–120 _(mmol/l·min)^1^	642 ± 165	534 ± 205	698 ± 219	613 ± 155	0.6	0.5
AUC insulin_0–120 _(nmol/l·min)	35 ± 13	29 ± 11	24 ± 8	24 ± 15	0.13	0.5
AUC insulin_0–120 _(nmol/l·min)^1^	20 ± 8	21 ± 10	15 ± 9	15 ± 13	0.3	0.4
ISI_0,120 _(mg·l^2^/mmol·mU·min)	44 ± 11	55 ± 21	36 ± 3	52 ± 19	0.11	0.8
HOMA2 %B	83 ± 36	67 ± 23	43 ± 11	49 ± 15	0.03	0.14
HOMA2 %S	54 ± 35	92 ± 30	65 ± 12	74 ± 25	0.4	0.3
HOMA2 IR	2.4 ± 1.0	1.3 ± 0.4	1.6 ± 0.3	1.5 ± 0.5	0.09	0.3

Baseline differences and carry-over effects between groups randomized to start with Paleolithic diet first or Diabetes diet first (mean ± SD). Carry-over effect tested for on individual means for both diets ((Paleolithic diet plus Diabetes diet)/2). *P for difference between groups with different starting diets in a two-sided t-test with independent samples, except for categorically reported values for sex, diabetic values at OGTT and drug treatment (e.g. lipid lowering drug), where P is for difference in Fisher's exact two-sided test with independent samples. ^1^stimulated secretion, area under curve at OGTT with value at 0 minutes as baseline.

One participant stopped taking sulfonylurea (glibencklamide 87.5 mg daily) the day after starting the study with the Paleolithic diet, and was thus on a low dose sulfonylurea at baseline, but without sulfonylurea during both the Paleolithic and Diabetes diet. Exclusion of this participant would not negate any significant effects from the Paleolithic diet compared to the Diabetes diet, but would negate the effect from the Paleolithic diet compared to baseline on systolic blood pressure, and the effect from the Diabetes diet compared to baseline on BMI. Due to concerns for rising blood sugar levels one participant switched hypertensive treatment from a thiazide diuretic to a beta blocker for seven weeks during the Paleolithic diet. Exclusion of this participant would not negate the significant effect on HbA1c, but would negate the effect on diastolic blood pressure. Due to concerns about muscle ache one participant was without lipid-lowering drug treatment for four weeks during the Paleolithic diet. Exclusion of this participant would not negate the significant effects on TG and HDL, but would instead cause also total cholesterol to be significantly lower following the Paleolithic diet compared to the Diabetes diet (*p *= 0.03). One participant was put on finasteride (5 mg daily, a drug versus benign prostate hyperplasia) during the Paleolithic diet and continued this medication during the following Diabetes diet.

### Baseline data

The group starting with the Paleolithic diet differed at baseline only with regard to fasting plasma glucose and AUC glucose being lower and HOMA2 %B being higher compared to the group starting with a Diabetes diet (Table 2). There was no difference between starting groups before or at the end of the study in inclusion/exclusion variables.

### Outcome variables

**Figure 1:**
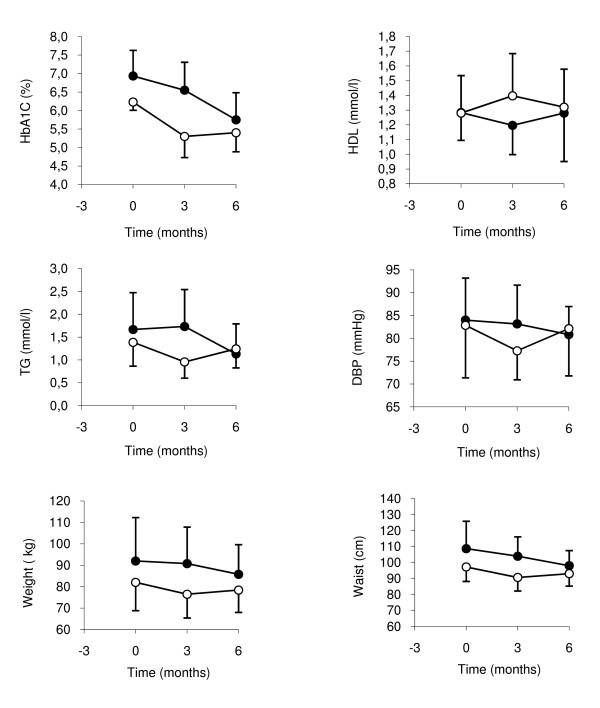
**Cardiovascular risk factors with significant effects from Paleolithic diet compared to Diabetes diet**. Closed circles depicts individuals starting with Diabetes diet first and open circles depicts individuals starting with Paleolithic diet first. Values are group means and error bars depicts SD for group means.

Compared to the Diabetes diet, the Paleolithic diet resulted in lower mean values of HbA1c, TG, DBP, weight, BMI and waist circumference, while mean values for HDL were higher (Table 3, Figure 1). The larger decrease of fasting plasma glucose following the Paleolithic diet nearly reached significance, and SBP also tended to decrease more following the Paleolithic diet. Compared to baseline, the Paleolithic diet lowered mean values of HbA1c, TG, SBP, weight, BMI, waist circumference, fasting plasma glucose, fasting plasma insulin, AUC glucose, ISI_0,120_, HOMA2 %S and HOMA2 %IR (Table 3). Compared to baseline, the Diabetes diet lowered mean values of BMI, waist circumference and HOMA2 %S (Table 3). Period effects were seen in AUC insulin_0–120_, AUC insulin_0–120 _stimulated secretion and HOMA2 %B (Table 4). Carry over effects were seen in HbA1c (Table 2, Figure 1).

**Table 3:** Risk factors for cardiovascular disease after Paleolithic diet and Diabetes diet (mean ± SD, Confidence Interval 95%)

	Paleolithic diet	P*	Diabetes diet	P**	Delta diets^1^	P***
HbA1C (%, Mono-S)	5.5 ± 0.7, 5.1 to 5.9	0.0001	5.9 ± 0.9, 5.5 to 6.4	0.001	-0.4	0.02
Cholesterol (mmol/l)	4.3 ± 1.2, 3.6 to 4.9	0.6	4.5 ± 1.2, 3.8 to 5.1	0.7	-0.2	0.3
LDL (mmol/l)	2.7 ± 1.0, 2.1 to 3.2	0.3	2.8 ± 1.1, 2.2 to 3.4	0.6	-0.1	0.5
HDL (mmol/l)	1.34 ± 0.30, 1.18 to 1.51	0.2	1.26 ± 0.23, 1.14 to 1.39	0.7	0.08	0.03
TG (mmol/l)	1.0 ± 0.5, 0.8 to 1.3	0.003	1.5 ± 0.7, 1.1 to 1.8	0.7	-0.4	0.003
CRP (mg/l)	2.0 ± 1.6, 1.1 to 2.9	0.2	2.6 ± 2.3, 1.4 to 3.8	0.8	-0.6	0.4
SBP (mmHg)	140 ± 12, 134 to 147	0.048	149 ± 22, 137 to 161	0.7	-8	0.13
DBP (mmHg)	79 ± 6, 76 to 82	0.06	83 ± 9, 78 to 88	0.7	-4	0.03
Weight (kg)	81 ± 13, 74 to 88	0.005	84 ± 15, 76 to 92	0.052	-3	0.01
BMI (kg/m^2^)	28 ± 5, 25 to 30	0.01	29 ± 6, 26 to 32	0.03	-1	0.04
Waist (cm)	94 ± 9, 89 to 99	0.01	98 ± 11, 92 to 104	0.02	-4	0.02
fP-glucose (mmol/l)	7.0 ± 1.4, 6.2 to 7.8	0.01	7.5 ± 1.4, 6.7 to 8.2	0.2	-0.5	0.08
fP-insulin (pmol/l)	69 ± 30, 53 to 85	0.02	67 ± 20, 57 to 78	0.06	2	0.8
AUC glucose_0–120 _(mmol/l·min)	1398 ± 314, 1227 to 1568	0.01	1478 ± 358, 1283 to 1672	0.09	-80	0.2
AUC glucose_0–120 _(mmol/l·min)^2^	558 ± 196, 452 to 665	0.09	582 ± 213, 467 to 698	0.2	-24	0.7
AUC insulin_0–120 _(nmol/l·min)	26 ± 14, 19 to 34	0.10	27 ± 13, 20 to 34	0.4	0	0.9
AUC insulin_0–120 _(nmol/l·min)^2^	18 ± 13, 11 to 25	0.9	19 ± 11, 12 to 25	0.8	-1	0.7
ISI_0,120 _(mg·l^2^/mmol·mU·min)	56 ± 22, 44 to 68	0.02	50 ± 20, 40 to 61	0.052	6	0.2
HOMA2 %B	63 ± 27, 48 to 77	0.6	55 ± 19, 44 to 66	0.2	8	0.2
HOMA2 %S	89 ± 45, 64 to 113	0.02	79 ± 23, 66 to 91	0.04	10	0.4
HOMA2 IR	1.4 ± 0.6, 1.1 to 1.7	0.01	1.4 ± 0.4, 1.1 to 1.6	0.052	0	0.9
Diabetic values at OGTT yes/no (n)	8/5	0.13	9/4	0.3	-1/1	1.0

Risk factors for cardiovascular disease after 3 months of Paleolithic diet compared to 3 months of Diabetes diet (mean±SD, Confidence Interval 95%). ^1^Mean value for Paleolithic diet minus mean value for Diabetes diet. All P-values from two-sided t-test  with dependent samples, except for Diabetic values at OGTT where P is for difference  between diets in Fischer's two-sided exact test with related samples. *P for difference  between Paleolithic diet and baseline, **P for difference between Diabetes diet and  baseline and ***P for difference between diets. ^2^Stimulated secretion, area under  curve at OGTT with value at 0 minutes as baseline.

**Table 4:** Period effects on cardiovascular risk factors after 3 and 6 months in all 13 subjects combined (mean ± SD)

	Baseline	3 months	6 months	P*
HbA1C (%, Mono-S)	6.6 ± 0.6	5.9 ± 0.9	5.6 ± 0.6	0.10
Cholesterol (mmol/l)	4.4 ± 1.1	4.4 ± 1.1	4.3 ± 1.3	0.6
LDL (mmol/l)	2.9 ± 0.9	2.8 ± 1.0	2.7 ± 1.0	0.5
HDL (mmol/l)	1.3 ± 0.2	1.3 ± 0.3	1.3 ± 0.3	0.9
TG (mmol/l)	1.5 ± 0.7	1.3 ± 0.7	1.2 ± 0.5	0.5
CRP (mg/l)	2.4 ± 1.8	2.8 ± 2.5	1.8 ± 1.0	0.14
SBP (mmHg)	150 ± 21	144 ± 16	145 ± 21	0.9
DBP (mmHg)	83 ± 8	80 ± 8	82 ± 8	0.4
Weight (kg)	87 ± 17	83 ± 15	82 ± 12	0.4
BMI (kg/m^2^)	30 ± 7	29 ± 6	28 ± 4	0.4
Waist (cm)	103 ± 14	96.8 ± 12	95 ± 9	0.5
fP-glucose (mmol/l)	7.8 ± 1.2	7.1 ± 1.6	7.3 ± 1.3	0.5
fP-insulin (pmol/l)	98 ± 44	74 ± 28	63 ± 21	0.2
AUC glucose (mmol/l·min)	1607 ± 218	1438 ± 350	1437 ± 329	1.0
AUC glucose (mmol/l·min)^1^	667 ± 186	583 ± 189	558 ± 219	0.6
AUC insulin (nmol/l·min)	30 ± 12	29 ± 12	23 ± 14	0.01
AUC insulin (nmol/l·min)^1^	18 ± 8	21 ± 11	16 ± 13	0.01
ISI_0,120 _(mg·l^2^/mmol·mU·min)	40 ± 9	50 ± 16	56 ± 25	0.2
HOMA2 %B	65 ± 34	65 ± 27	53 ± 18	0.04
HOMA2 %S	59 ± 27	80 ± 42	87 ± 28	0.6
HOMA2 IR	2.0 ± 0.8	1.5 ± 0.6	1.3 ± 0.4	0.2
Diabetic values at OGTT yes/no (n)	12/1	8/5	9/4	1.0

*P for difference between 3 and 6 months in a two-sided t-test with dependent samples, except for Diabetic values at OGTT where P is for difference between diets in Fischer's two-sided exact test with related samples. ^1^stimulated secretion, area under curve at OGTT with value at 0 minutes as baseline.

### Reported food intake

There were no period or carry-over effects in reported dietary intakes (data not shown). Reported daily food intake differed between diets mainly in that the Paleolithic diet was markedly lower in cereals and dairy products, and lower in potatoes, beans and bakery, and much higher in fruits, vegetables, meat and eggs (Table 5). Further, the Paleolithic diet was somewhat lower in total energy, energy density, carbohydrate, fiber, saturated fatty acids and calcium, and higher in monosaccharides, dietary cholesterol, some vitamins (vitamin B6, vitamin C, niacin) and minerals (potassium, selenium) (Table 5). During the Paleolithic diet, there was a lower relative intake (as a percentage of total macronutrient energy intake [E%]) of carbohydrate and a higher relative intake of protein and fat (Table 5). Both dietary GL and dietary GI were determined to be lower for the Paleolithic diet than for the Diabetic diet (Table 5).

**Table 5:** Average food eaten per day during Paleolithic diet and Diabetes diet (mean ± SD)

	Paleolithic	Diabetes	P*
Total weight (g)	1445 ± 367	1456 ± 312	0.9
Total energy (MJ)	6.6 ± 1.2	7.9 ± 1.6	0.005
(kcal)	1581 ± 295	1878 ± 379	0.005
Energy density (kJ/g)	4.7 ± 0.7	5.6 ± 1.1	0.02
Protein (g)	94 ± 18	90 ± 14	0.5
(E%)	24 ± 3	20 ± 4	0.0001
Carbohydrate (g)	125 ± 43	196 ± 61	0.00001
(E%)	32 ± 7	42 ± 7	0.0001
Fat (g)	68 ± 11	72 ± 20	0.6
(E%)	39 ± 5	34 ± 6	0.04
Alcohol (g)	6.3 ± 8.9	3.6 ± 5.6	0.2
(E%)	3 ± 4	1 ± 2	0.08
Fiber (g)	21 ± 8	26 ± 8	0.02
(E%)	2.5 ± 0.7	2.7 ± 0.7	0.4
Glycemic Load (g)	63 ± 23	111 ± 41	0.00002
Glycemic Index	50 ± 5	55 ± 6	0.01
Monosaccharides (g)	46 ± 21	33 ± 16	0.03
Disaccharides(g)	31 ± 14	39 ± 15	0.10
Sucrose (g)	29 ± 13	30 ± 12	0.8
Saturated fatty acid (g)	19 ± 5	27 ± 9	0.002
Monounsaturated fatty acid(g)	30 ± 6	26 ± 7	0.13
Polyunsaturated fatty acid (g)	14 ± 4	12 ± 4	0.2
Fatty acid C4:0-C10:0 (g)	0.3 ± 0.4	2.1 ± 1.3	0.0001
Fatty acid C12:0 (g)	0.3 ± 0.3	1.1 ± 0.8	0.002
Fatty acid C14:0 (g)	1.3 ± 0.5	2.8 ± 1.3	0.0002
Fatty acid C16:0 (g)	12 ± 3	14 ± 4	0.02
Fatty acid C16:1 (g)	2.0 ± 0.5	1.5 ± 0.6	0.03
Fatty acid C18:0 (g)	4.5 ± 1.5	5.9 ± 1.9	0.053
Fatty acid C18:1, oleic acid (g)	26 ± 6	24 ± 7	0.3
Fatty acid C18:2, n-6, Linoleic acid (g)	9 ± 4	8 ± 3	0.6
Fatty acid C18:3, n-3, ALA (g)	1.5 ± 0.7	1.6 ± 0.8	0.6
Fatty acid C20:0 (g)	0.1 ± 0.1	0.1 ± 0.1	0.2
Fatty acid C20:4, n-6 (g)	0.2 ± 0.1	0.1 ± 0.1	0.01
Fatty acid C20:5, n-3, EPA (g)	0.6 ± 0.3	0.3 ± 0.3	0.052
Fatty acid C22:5, n-3 (g)	0.2 ± 0.1	0.1 ± 0.1	0.3
Fatty acid C22:6, n-3, DHA (g)	1.3 ± 0.7	0.7 ± 0.7	0.06
Cholesterol (mg)	577 ± 107	365 ± 88	0.0003
Vitamin A, Retinolequivalents (μg)	896 ± 534	1139 ± 450	0.2
Vitamin A, Retinol (μg)	385 ± 333	673 ± 353	0.051
Vitamin A, Caroten (μg)	5038 ± 3414	4811 ± 5633	0.9
Vitamin D (μg)	9 ± 4	9 ± 7	0.9
Vitamin E (mg)	13 ± 4	11 ± 3	0.07
Vitamin E, Alpha-tocopherol (mg)	13 ± 4	11 ± 3	0.07
Vitamin B-1, Thiamin (mg)	1.5 ± 0.5	1.6 ± 0.5	0.8
Vitamin B-2, Riboflavin (mg)	1.6 ± 0.3	1.6 ± 0.2	0.5
Vitamin B-6 (mg)	3.2 ± 0.7	2.4 ± 0.6	0.003
Vitamin B-12 (μg)	8.6 ± 4.0	6.7 ± 2.4	0.2
Vitamin B, Folate (μg)	340 ± 172	300 ± 79	0.4
Vitamin C, Ascorbic acid (mg)	219 ± 136	119 ± 60	0.03
Niacinequivalents (mg)	45 ± 11	39 ± 8	0.08
Niacin (mg)	27 ± 8	22 ± 6	0.03
Phosphorus (mg)	1233 ± 247	1437 ± 208	0.02
Iron (mg)	12 ± 3	12 ± 3	1.0
Potassium (mg)	3669 ± 982	3181 ± 908	0.0497
Calcium (mg)	356 ± 102	698 ± 220	0.00002
Magnesium (mg)	307 ± 84	311 ± 68	0.9
Sodium (mg)	2530 ± 924	2963 ± 678	0.14
Selenium (μg)	81 ± 20	55 ± 18	0.001
Zinc (mg)	11 ± 3	12 ± 2	0.3
Ash (g)	17 ± 4	19 ± 4	0.13
Water (g)	1113 ± 306	1049 ± 258	0.5
Fruits (g)	451 ± 200	251 ± 210	0.005
Vegetables (g)	346 ± 179	241 ± 176	0.0497
Potatoes (g)	49 ± 51	106 ± 84	0.03
Nuts (g)	29 ± 24	12 ± 20	0.13
Meat (g)	139 ± 67	73 ± 29	0.003
Meat products (g)	97 ± 76	71 ± 43	0.2
Fish (g)	104 ± 55	89 ± 56	0.5
Eggs (g)	71 ± 27	27 ± 24	0.001
Beans (g)	4 ± 14	24 ± 33	0.03
Cereals without rice (g)	11 ± 24	172 ± 96	0.00004
Rice (g)	7 ± 17	6 ± 10	0.9
Milk/milk products (g)	16 ± 32	183 ± 123	0.0002
Oil (g)	0.3 ± 0.7	1.4 ± 3.5	0.3
Sauce (g)	13 ± 20	30 ± 36	0.2
Bakery (g)	10 ± 18	34 ± 35	0.005
Jam (g)	0 ± 0	12 ± 22	0.07
Spirits (g)	1.0 ± 2.8	1.4 ± 4.1	0.8
Wine (g)	52 ± 83	20 ± 49	0.14
Beer (g)	31 ± 103	55 ± 80	0.4
Sweet beverages (g)	0 ± 0	38 ± 64	0.051
Juice (g)	12 ± 35	10 ± 26	0.6

Average food eaten per day during Paleolithic diet and Diabetes diet (mean ± SD). Estimated from 4 day weighed food records. *P for difference between diets in a two-sided t-test with dependent samples. E% = percent energy from total macronutrient energy.

### Exploratory analyses

In exploratory analyses of primary endpoints, within-subject differences (Δ) in HbA1c (ΔHbA1c) correlated with Δwaist circumference, which correlated with Δweight, which correlated with ΔCRP (Table 6). Furthermore, ΔHDL correlated with Δcholesterol and ΔDBP with ΔHOMA2 IR (Table 6). In exploratory analyses of estimated intake of nutrients, ΔHbA1c correlated with Δpotassium, ΔHDL with Δfatty acid C20:5 n-3, ΔTG with Δthiamin, ΔDBP with Δdietary cholesterol, Δweight with Δenergy density per meal, and Δwaist circumference with Δbakery, Δenergy density per meal, Δsauce and Δvitamin E (Table 6).

**Table 6:** Exploratory analyses

	Bivariate correlation P*	Pearson correlation r	Linear regression P^§^	Adjusted R^2^
ΔHbA1C versus outcome variables				
ΔWaist circumference (cm)	0.03	0.6	0.03	0.31
ΔHbA1C versus dietary variables				
ΔPotassium (mg)	0.03	0.6	0.03	0.32
ΔFruits (g)	0.04	0.6	NS	
ΔSauce (g)	0.04	-0.6	NS	
ΔMilk/milk products (g)	0.05	0.6	NS	
ΔHDL versus outcome variables				
ΔCholesterol (mmol/l)	0.03	0.6	0.03	0.32
ΔHDL versus dietary variables				
ΔFatty acid C20:5, n-3, EPA (g)	0.004	-0.7	0.004	0.51
ΔVitamin C, Ascorbic acid (mg)	0.02	-0.6	NS	
				
ΔTG versus outcome variables				
No correlations				
ΔTG versus dietary variables				
ΔVitamin B-1, Thiamin (mg)	0.03	0.6	0.03	0.29
				
ΔDBP versus outcome variables				
ΔHOMA2 IR	0.004	-0.7	0.01	0.67
ΔSBP (mmHg)	0.03	0.6	0.03	
ΔfP-insulin (pmol/l)	0.004	-0.7	NS	
ΔHOMA2 %S	0.01	0.7	NS	
ΔAUC insulin_0–120 _(nmol/l·min)	0.01	-0.7	NS	
ΔDBP versus dietary variables				
ΔDietary cholesterol (mg)	0.001	0.8	0.001	0.60
ΔFatty acid C16:1 (g)	0.002	0.8	NS	
ΔFatty acid C22:6, n-3, DHA (g)	0.01	0.7	NS	
ΔFatty acid C20:5, n-3, EPA (g)	0.02	0.6	NS	
ΔEggs (g)	0.03	0.6	NS	
				
ΔWeight versus outcome variables				
ΔCRP (mg/l)	0.000005	0.9	0.001	0.85
ΔBMI (kg/m^2^)	0.00002	0.9	NE	
ΔWaist circumference (cm)	0.0001	0.9	NS	
ΔISI_0,120 _(mg·l^2^/mmol·mU·min)	0.001	-0.8	NS	
ΔWeight versus dietary variables				
ΔEnergy density per meal (kJ/g)	0.00003	-0.9	0.00003	0.79
ΔVitamin E (mg)	0.01	-0.7	NS	
ΔVegetables (g)	0.01	0.7	NS	
ΔFatty acid C18:1 (g)	0.02	-0.6	NS	
				
ΔBMI versus outcome variables				
ΔWeight (kg)	0.00002	0.9	NE	
ΔWaist circumference (cm)	0.001	0.8	0.001	0.63
ΔCRP (mg/l)	0.001	0.8	NS	
ΔISI_0,120 _(mg·l^2^/mmol·mU·min)	0.003	-0.8	NS	
ΔBMI versus dietary variables				
ΔEnergy density per meal (kJ/g)	0.0004	-0.8	0.0004	0.67
ΔVitamin E (mg)	0.004	-0.7	NS	
ΔVegetables (g)	0.01	0.7	NS	
ΔFatty acid C18:1 (g)	0.01	-0.7	NS	
ΔFat (g)	0.02	-0.6	NS	
ΔFatty acid C18:2, n-6, Linoleic acid (g)	0.047	-0.6	NS	
				
ΔWaist circumference versus outcome variables				
ΔWeight (kg)	0.0001	0.9	0.0001	0.74
ΔBMI (kg/m^2^)	0.001	0.8	NS	
ΔCRP (mg/l)	0.001	0.8	NS	
ΔISI_0,120 _(mg·l^2^/mmol·mU·min)	0.002	-0.8	NS	
ΔHbA1C (%)	0.03	0.6	NS	
				
ΔWaist circumference versus dietary variables				
ΔBakery (g)	0.01	-0.7	0.006	0.92
ΔEnergy density per meal (kJ/g)	0.001	-0.8	0.007	
ΔSauce (g)	0.02	-0.6	0.009	
ΔVitamin E (mg)	0.003	-0.7	0.03	
ΔFatty acid C18:1 (g)	0.01	-0.7	NS	
ΔVitamin E, Alpha-tocopherol (mg)	0.04	-0.6	NS	

Exploratory analyses performed on outcome variables with significant effects from Paleolithic diet compared to Diabetes diet. Analyses consisted of bivariate correlations between within-subject differences (Δ) in outcome and dietary variables. Significantly correlating variables were entered into a stepwise forward linear regression analyses. *P for bivariate correlation between variables in a two-sided t-test. §P for stepwise forward linear regression analyses entering significantly correlated variables. NS = Not Significant. NE = Weight and BMI not entered in their respective regression analysis due to calculatory relatedness.

## Discussion

### Key findings

The advice for patients with type 2 diabetes to follow a Paleolithic diet resulted in lower HbA1c, TG, DBP, weight and waist circumference, and higher HDL, as compared to a Diabetes diet according to current guidelines. In addition, fasting glucose and SBP tended to decrease more after the Paleolithic diet. Changes in glucose tolerance were not significantly different between diets. The two diets differed mainly in that the Paleolithic diet was lower in cereals and dairy products, and higher in fruits, vegetables, meat and eggs. Further, the Paleolithic diet was lower in total energy, energy density, carbohydrate, dietary GL, saturated fatty acids and calcium, and higher in unsaturated fatty acids, dietary cholesterol and several vitamins. Dietary GI was lower in the Paleolithic diet (GI = 50) than in the Diabetic diet (GI = 55).

### Possible mechanisms and explanations

No advice was given to restrict food intake. Therefore, the lower reported energy intake during the Paleolithic diet despite no difference in weight of reported food intake agrees with the notion that such a diet is satiating and facilitates a reduced caloric intake [4,27]. Accordingly, energy density was lower in the Paleolithic diet and also correlated with alterations of both weight and waist circumference. The higher amount of fruit and vegetables during the Paleolithic period may have promoted weight loss due to its high content of water, which is thought to be satiating [28]. Interestingly, the Paleolithic diet appeared to be satiating despite a lower content of fiber in this study. The slightly higher relative protein intake, as percentage of total calorie intake, may also have added to a satiating effect [29,30]. Alternative explanations on satiation, such as dietary effects on leptin resistance, could also be considered [31].

A reduced energy intake would evidently be a major explanation for the beneficial effects of the Paleolithic diet on weight and waist circumference. Meta-analyses and large trials with various lifestyle interventions indicate that reduced caloric intake is more important for long-term weight loss than other known dietary factors, including macronutrient composition [32-40]. In studies shorter than 6 months, such as this one, differences in GI and/or GL may also have played a role for weight change. A Cochrane review found that overweight or obese people lost slightly more weight during 5–12 weeks of low GI diets [41], and short-term carbohydrate restriction possibly results in greater weight loss than low-fat diets [29]. However, dietary GI and dietary GL did not correlate with alterations of weight, waist circumference or metabolic variables in our study. It should also be noted that, in the present study, reported mean absolute carbohydrate intake in the Paleolithic diet (g per day) was only slightly below the 130 g per day recommended by the American Diabetes Association, and clearly above 50 g per day, which has been proposed as the level below which a diet should be termed a low carbohydrate diet [42].

Paleolithic diet improved the glycemic control in the subjects, as evident by the reduction of HbA1c levels by -0.4 percentage points lower as compared to the diabetes diet. Since both glucose and insulin levels declined during Paleolithic diet, a main mechanism behind the improved glycemic control is probably improved insulin sensitivity, which may have allowed the released insulin to work more efficiently. The difference in reduction in HbA1c of 0.4% units between the Paleolithic and Diabetes diet is close to the average 0.5% units in a recent Cochrane review of diets with a low glycemic index or glycemic load [8]. However, the differences in GI between diets in that meta-analysis were considerably larger than in our trial. Glucose tolerance, which also determines the glucose response and thereby HbA1c, did not improve more during the Paleolithic diet. This result agrees with findings from Frassetto et al [10], but differs from our previous parallel-group trial which compared a Paleolithic diet with a Mediterranean-like diet in subjects with diabetes or impaired glucose tolerance [4]. Glucose tolerance has not been shown to improve after reduced carbohydrate intake in earlier dietary studies [43-46].

The much higher fruit intake of the Paleolithic diet probably resulted in a slightly higher intake of fructose which may have aided in the reduction of HbA1c. Fructose in exchange for starch, sucrose or glucose decreases postprandial glycemia [47], while the effect on glucose tolerance and insulin sensitivity is more uncertain [48]. The effect of fruit on TG and other risk factors is expected to have been neutral in this study [48,49]. Total intake of monosaccharides was 46 g per day, including approximately equal amounts of glucose and fructose, which was well below the suggested safety limit of 50 g fructose per day [48]. Our study lends further support to the notion that fruit intake should not be restricted in patients with type 2 diabetes.

The lower DBP after the Paleolithic diet compared to the Diabetes diet did not correlate with sodium intake, which did not differ significantly and was rather low in both diets (2.5 g and 3.0 g per day respectively for the Paleolithic and Diabetes diet).

The reduction of TG after the Paleolithic diet was possibly due to greater loss of abdominal fat [50] or lower GL compared to the Diabetes diet [6], although no correlation of TG with waist loss or GL was seen in exploratory analyses. A small additional effect on TG may be attributable to a trend for higher content of long-chain omega-3 fatty acids in the Paleolithic diet, while the higher dietary cholesterol content of the Paleolithic diet is probably of minor significance [51].

### Comparison with findings from other studies

All improvements in markers of the metabolic syndrome on the Paleolithic diet are in line with findings from epidemiological studies in non-Western populations [14-16]. Improvements in HbA1c [4], weight [4,12,52], BMI [52], waist circumference [4,52], DBP [10], and TG [10], compared to baseline, on a Paleolithic diet have been observed before in intervention studies, while improvements in HDL have not. Similar differences in weight and DBP on a Paleolithic diet, compared to a cereal based diet, have been observed before in an intervention study on domestic pigs [11]. A lower reported energy intake and energy density of food despite food intake ad libitum agrees with our earlier findings that a Paleolithic diet facilitates a reduction of caloric intake [4,11,27].

Also, lower intake of cereals, dairy products, carbohydrates, dietary GL and saturated fat, and higher intake of fruit and potassium have been observed before [4,10]. Lower intake of potatoes, bakery, fiber, phosphorous and calcium, and higher intake in vegetables, meat, eggs, monosaccharides, dietary cholesterol, vitamin B6, vitamin C, niacin and selenium have not been observed before in intervention studies with a Paleolithic diet. Dietary GI for a Paleolithic diet has not been determined before.

### Limitations of the present study

A limitation of this study, as with most other dietary trials, is the lack of blinding after randomization. To minimize this problem, all study participants were informed of the intention to compare two healthy diets in the treatment of type 2 diabetes and that it was unknown if any of them would be superior to the other. Also, written information with dietary advice and food recipes were similarly formulated for both diets. Furthermore, for increased conformity, the dietary advice and data collection procedure were discussed by all those administering the interventions at several meetings prior to start of study.

Another limitation of this study is its small size which did not reach the number of participants needed as calculated in the pre-study power calculation. The decision to end the study was taken when recruitment for the study had not yielded new participants for more than six months. The population of patients with type 2 diabetes is much larger and therapy continues for substantially longer than in this study. Moreover, many patients with type 2 diabetes have illnesses and treatments that excluded them from the current study. Consequently, the results of this study do not address the occurrence of rare adverse events, nor can they be extrapolated to all patients seen in general clinical practice.

The carry-over effects on HbA1c were not due to carry-over or period effects in reported food intake. Instead, they could be true carry-over effects of the first diet. This is particularly likely for HbA1c, since HbA1c represents a weighted average of the blood glucose concentration over the previous two to three months ([53]. If results from the second period were discarded (owing to carryover [54]), the reduction of HbA1c from the Paleolithic diet compared to the Diabetes diet was still significant (*p *= 0.01) and even larger (-1.3% units) than when results from the second period were included. However, this approach could lead to biased answers to our hypothesis and results from both periods are therefore used in this study [54].

The lack of carry-over or period effects in reported food intake indicates fairly good adherence to intervention diets. Reported food intake in this study seemed reasonable both in distribution and quantity, as subjectively assessed by a nutrition engineer skilled in analyzing reported food intake (YG). Furthermore, the reported lower energy intake of 1.3 MJ per day on a Paleolithic diet equals about 3.2 kg fat during three months, which almost exactly accounts for the observed 3.3 kg difference in weight loss between diets. This indicates both good reporting by the participants and good adherence to reported food intake during the study.

### Clinical and research implications

The favourable results in this study are in line with previous findings and increase the generalizability of the Paleolithic diet by testing it in both men and women in a primary care setting. A limitation of the study is the small size of the study population. This prevents the conclusions from resulting in nutritional recommendations for patients with type 2 diabetes. A long-term study in a larger population is therefore required. In parallel, further research into possible mechanisms for the beneficial effects of a Paleolithic diet should be done.

Total protein intake in g per day did not differ between the diets, but, as a result of the difference in total energy intake, the energy percentage (E%) from dietary protein on the Paleolithic diet (24 E%) slightly exceeded US and European recommendations for people with diabetes (<20 E%) [17,55]. The debatable disadvantage for long-term kidney function [56,57] should be weighed against the benefits of attenuated postprandial glycemia when protein replaces starch or glucose [58].

Calcium intake did not meet recommendations for any of the diets, and it was particularly low in the Paleolithic diet. Recent calcium balance studies indicate that human calcium requirements are lower than previously thought [59], and meta-analyses of randomized controlled trials suggest that the effect of calcium supplementation for bone strength is limited [60,61]. It has been suggested that absorption and excretion of calcium are more important than calcium intake for whole-body calcium balance [62]. In this context, the lower content of calcium-binding phytate and the lower dietary acid load from a Paleolithic diet may hypothetically compensate for the low amount of calcium [63]. Supporting this view are the findings of Frassetto et al, where calcium intake remained unchanged and urine calcium decreased after a Paleolithic diet compared to baseline [10].

As has been discussed, there may be a challenge to implement and adopt the Paleolithic diet on a worldwide scale in subjects with type 2 diabetes. However, this aspect is beyond the objective of this paper and requires more research.

## Conclusion

Based on the results of this 3-month randomized cross-over study in subjects with type 2 diabetes, a Paleolithic diet improves glycemic control in association with improvement of several cardiovascular risk factors compared to a conventional diabetes diet. The study supports the initiation of a large scale study on the effect of Paleolithic diet in subjects with type 2 diabetes.

## Competing interests

The authors declare that they have no competing interests.

## Authors' contributions

TJ participated in the design and execution of the study, participated in statistical analysis, and conceived of and wrote the article. YG analyzed reported food intake and participated in the design of the article as well as revising it for important intellectual content. BA participated in the design of the study, carried out the analysis of glucose and insulin in OGTTs, and revised the article for important intellectual content. UCB, GP, AH and MS participated in the design and execution of the study, and revised the article for important intellectual content. SL participated in the design of the study, participated in statistical analysis, and participated in the design of the article as well as revising it for important intellectual content. All authors read and approved the final manuscript.
